# Antioxidant Potential and Characterization of Polyphenol Compounds in *Moringa oleifera* Pods

**DOI:** 10.1002/fsn3.4628

**Published:** 2024-12-01

**Authors:** Rongjia Xie, Eric N. Ponnampalam, Farhad Ahmadi, Frank R. Dunshea, Hafiz A. R. Suleria

**Affiliations:** ^1^ School of Agriculture, Food and Ecosystem Sciences, Faculty of Science The University of Melbourne Parkville Victoria Australia; ^2^ Agrifeed Animal Production Victoria Australia; ^3^ Faculty of Biological Sciences The University of Leeds Leeds UK; ^4^ Centre for Sustainable Bioproducts Deakin University Waurn Ponds Victoria Australia

**Keywords:** antioxidant activity, nutritional composition, polyphenol characterization

## Abstract

The aim of this investigation was to comparatively assess the antioxidant and polyphenol compounds in fresh moringa pods sourced from two different regions in Australia, namely Queensland (QLD) and Western Australia (WAU). Total polyphenol content varied between 1.64 and 5.97 mg GAE/g in moringa pod samples from QLD, while it ranged from 2.84 to 4.31 mg GAE/g in WAU samples. Total flavonoid content in QLD and WAU samples averaged 4.62 and 4.24 mg QE/g, respectively. Total condensed tannin content in QLD and WAU samples averaged 2.07 and 1.60 mg CE/g, respectively. The QLD samples had higher DPPH (2.87 vs. 2.74 mg AAE/g), ABTS (15.0 vs. 12.9 mg AAE/g), and total antioxidant capacity (2.34 vs. 1.46 mg AAE/g) than WAU samples. LC‐ESI‐QTOF‐MS/MS analysis identified 111 polyphenol compounds in moringa pod samples, including phenolic acids, flavonoids, and tannins. Some compounds were prevalent across most samples, such as 3‐sinapoylquinic acid and theaflavin. The study revealed that moringa pods contain a high concentration of polyphenols with strong antioxidant capacity. These findings highlight the substantial influence of regional effects on the polyphenol content and bioactive properties of moringa pods.

## Graphical abstract

This study examines the influence of environmental conditions on the antioxidant potential of fresh moringa pods cultivated in two Australian states. The pods are a rich source of polyphenols with potent antioxidant properties.

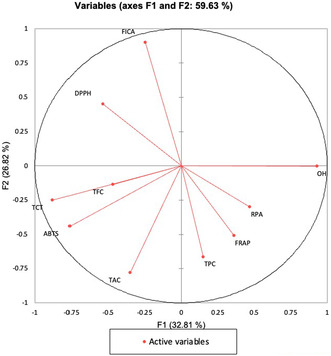

**Abbreviations**
ABTS: 2,2′‐azinobis‐(3‐ethylbenzothiazoline‐6‐sulfonic acid)
DPPH: 2,2′‐diphenyl‐2‐picrylhydrazyl
FICA: ferrous ion chelating activity
FRAP: ferric reducing antioxidant power
LC‐ESI‐QTOF‐MS/MS: liquid chromatography‐electrospray ionization quadrupole time‐of‐flight mass spectrometry
QLD: samples from Queensland
RPA: reducing power assay
TAC: total antioxidant capacity
TFC: total flavonoid content
TCT: total condensed tannin
TPC: total polyphenol content
WAU: samples from Western Australia
•OH‐RSA: hydroxyl radical scavenging activity

## 1 Introduction

Moringa is commonly grown in subtropical and tropical regions and is known for its significant nutritional and therapeutic benefits (Du, Wu et al. 2021). Its leaves, pods, and seeds are used as food, pharmaceuticals and cosmetics for their health benefits (Ogunsina, Radha, and Govardhan Singh 2010; Xu, Chen, and Guo 2019). In recent years, moringa has gained attention in scientific research due to its remarkable nutritional profile and its potential as a source of pharmacological compounds.

Polyphenol compounds represent a class of chemical constituents recognized for their bioactive potential. The spectrum of polyphenol compounds in moringa is particularly diverse and includes an array of substances such as flavonoids and phenolic acids, including gallic acid, vanillin, kaempferol, chlorogenic acid, myricetin, quercetin, luteolin, and rutin (Lin, Zhang, and Chen 2018). These compounds are indispensable for their role in fortifying the plant against environmental challenges and their significant contributions to human health (Al Juhaimi, Ghafoor, Ahmed et al. 2017). The complex structures of these polyphenol compounds facilitate a wide range of biological activities, conferring protective benefits against oxidative stress and cellular damage (Athira et al. 2021; Vonghirundecha et al. 2022). Moringa is rich in polyphenols, including flavonoids, phenolic acids, and tannins, which are important for their antioxidant properties. These components can scavenge and neutralize free radicals, which are volatile molecules that may disrupt cellular function, accelerate the aging process, and potentially trigger some diseases (Zhu, Yin, and Yang 2020).

Although considerable research has been undertaken on the composition of moringa, there is still a crucial need for further investigation into its antioxidant potential under different environmental conditions, such as climate and geographical location which may influence the composition and concentration of bioactive compounds in moringa pods, leaves or seeds. Existing studies indicate variations in the nutritional and phytochemical attributes of moringa, contingent upon factors such as climate, altitude, and soil type (Iqbal and Bhanger 2006; Kim et al. 2021). The antioxidant activity of moringa may be affected by extraction methods, crop conditions, harvest time, and storage conditions (Vázquez‐León et al. 2017). The plant age, environmental conditions, and the specific parts of the plant collected may significantly alter the amount of polyphenol compounds and their antioxidant (Al Juhaimi, Ghafoor, Babiker et al. 2017; Qadir et al. 2022). Previous investigations have predominantly concentrated on moringa leaves and seeds' biochemical properties and health benefits. The pods of moringa are a notable source of polyphenol compounds with potent antioxidant properties. Despite this, research comparing the polyphenol content and bioactivity of moringa cultivated in different regions of Australia remains limited. Thus, this study was designed to assess the antioxidant activities and polyphenol characterization of moringa pods collected from two Australian states: Queensland and Western Australia.

## 2 Materials and Methods

### 2.1 Chemical and Reagents

Vanillin, gallic acid, Folin‐Ciocalteu reagent, L‐ascorbic acid, sodium phosphate, iron chloride hexahydrate, hexahydrate aluminium chloride, hydrated sodium acetate, ammonium molybdate, hydrochloric acid, sodium carbonate anhydrous, catechin, quercetin, 2,4,6‐tripyridyl‐s‐triazine (TPTZ), 2,2′‐diphenyl‐2‐picrylhydrazyl (DPPH), and 2,2′‐azinobis‐(3‐ethylbenzothiazoline‐6‐sulfonic acid) (ABTS) were procured from Chem‐Supply Ltd. (Melbourne, VIC, Australia). Sulfuric acid (H_2_SO_4_) was sourced from RCI Labscan Ltd. (Bangkok, Thailand). Caffeic acid, *p*‐hydroxybenzoic acid, caftaric acid, protocatechuic acid, sinapinic acid, chlorogenic acid, syringic acid, ferulic acid, coumaric acid, quercetin‐3‐galactoside, diosmin, quercetin‐3‐glucuronide, quercetin‐3‐glucosidekaempferol, kaempferol‐3‐glucoside, and epicatechin gallate were acquired from Sigma‐Aldrich (Castle Hill, NSW, Australia). The reagents used for liquid chromatography‐mass spectrometry (LC‐MS/MS), including acetonitrile, methanol, ethanol, glacial acetic acid, and formic acid, were sourced from Thermo Fisher Scientific Inc. (Scoresby, Victoria, Australia).

### 2.2 Sample Preparation and Extraction

Samples of moringa pods were collected from two different regions (Queensland [QLD] and Western Australia [WAU]) in Australia: Queensland (QLD1, QLD2, QLD3, QLD4, QLD5, QLD6, QLD7) and Western Australia (WAU5, WAU7, WAU10, WAU15, WAU17, WAU20, and WAU32). The moringa plants belong to the PKM variety, and pods are used for human consumption. Pods were collected as they were available in QLD and WAU. Pods from Queensland and Western Australia were collected in December 2022 and April 2023, respectively. In farms from both regions, seven trees were randomly chosen, and seven young pods were randomly collected from each tree. Pods collected from each tree were separately wrapped in a polythene bag, and all bags containing fresh pods were packed in a large ice chest with ice packs. Fresh pods were taken directly via flight to Melbourne, Victoria. The following day, they were taken to the University of Melbourne's Food laboratory, and each fresh pod was cut in half. One portion of each half fresh pods was further cut into small cubes (~1 × 1 × 1 cm^3^), weighed, and dried at 60°C for 72 h. Upon recording the dry weights, samples from each tree were separately ground using a UDY Cyclone grinder (Fort Collins, CO, USA) fitted with a 1 mm mesh screen and stored in airtight plastic containers. All ground samples of fresh pods with the containers were kept in dark, refrigerated conditions. The remaining half of each pod was wrapped in separate polythene bags for each tree and maintained under frozen conditions for 3 months, which were not used for this study.

From all ground samples prepared using fresh pods (halves), homogeneous samples were used to analyze polyphenol compounds and antioxidant activity. The extraction process involved mixing 1 g of the sample with a solution comprising ethanol (70%) and formic acid (0.1%). Then, the samples underwent homogenization (10,000 rpm for 30 s) using a homogenizer (Staufen, IKA, Germany). The process continued with a 16‐h incubation period at 10°C and a shaking speed of 120 rpm, utilizing a shaking incubator (Ashwood, VIC, Australia). Afterwards, the samples underwent centrifugation at 4°C and 8000 rpm for 15 min (Tuttlingen, BW, Germany), and filtered through a 0.22 μm nylon membrane filter (Thermo Fisher Scientific Inc., USA). The supernatant was carefully collected and stored at −20°C, awaiting subsequent analysis. For LC‐MS/MS analysis, the extract was filtered through a syringe filter with a pore size of 0.45 μL.

### 2.3 Polyphenol Content and Antioxidant Activity

The modified methodologies from Gu et al. (2019) and Suleria, Barrow, and Dunshea (2020) were used for quantification of polyphenol compounds. Seven antioxidant assays, including ferric reducing antioxidant power (FRAP), DPPH, ABTS, total antioxidant capacity (TAC), reducing power assay (RPA), hydroxyl radical scavenging activity (^•^OH‐RSA), and ferrous ion chelating activity (FICA), were used in this investigation. All assays were conducted in triplicates by Multiskan Go microplate photometer (Waltham, MA, USA). Standard curves were generated with an R‐squared value exceeding 0.995.

#### 2.3.1 Total Phenolic Acid Content (TPC) Assessment

The TPC was determined spectrophotometrically as per the methodology reported by Samsonowicz, Regulska (Samsonowicz et al. 2019). A mixture of 25 μL of the extracts, 25 μL of Folin–Ciocalteu reagent (diluted 1:3 with water), and 200 μL of water were added to a 96‐well plate. Samples were incubated at 25°C for 5 min, with an additional 1‐h incubation at the same temperature after adding 25 μL 10% (w/w) sodium carbonate. Absorbance was measured (Waltham, MA, USA) at 765 nm. Varying concentrations of gallic acid, ranging from 0 to 200 μg/mL were used for standard curve creation.

#### 2.3.2 Total Flavonoid Content (TFC) Assay

The aluminum chloride method reported by Stavrou, Christou, and Kapnissi‐Christodoulou (2018) was used for TFC measurement. A mixture of 2% aluminum chloride (80 μL) and 50 g/L sodium acetate (120 μL) was added to a 96‐well plate. The mixture was allowed to incubate for 2.5 h at 25°C. Absorbance was read at 440 nm. A standard curve was constructed using a methanolic solution of quercetin (with concentrations ranging from 0 to 50 μg/mL) and presented as mg quercetin equivalents (QE)/g of sample.

#### 2.3.3 Total Condensed Tannin (TCT) Assay

Quantification of TCT was undertaken according to the methodology reported by Peng et al. (2019) with some modifications. In brief, 25 μL of the extract and 150 μL 4% (w/v) vanillin solution were mixed, followed by the addition of 25 μL sulfuric acid (32%). The solution was incubated at room temperature for 15 min. Absorbance was read at 500 nm. A standard curve was created using a catechin solution with concentrations between 0 and 1000 μg/mL. The results were reported as mg catechin equivalents (CE)/g of the sample.

#### 2.3.4 DPPH Evaluation

The DPPH activity was determined using the procedure of Vella, Cautela, and Laratta (2019). In brief, the extract (40 μL) was mixed with 0.1 M DPPH solution in methanol (260 μL) and allowed to incubate at 25°C for 30 min. Absorbance was measured at 517 nm. A standard curve was created by employing various doses of ascorbic acid dissolved in an aqueous solution, with a range of 0–50 μg/mL. The results were presented as ascorbic acid equivalent (AAE)/g of the sample.

#### 2.3.5 Ferric Reducing Antioxidant Power Assay

The FRAP assay was undertaken using a method outlined by Sogi et al. (2013), with slight modifications. In brief, the FRAP reagent was freshly made by combining a 300 mM solution of sodium acetate (with a pH of 3.6), a 10 mM solution of TPTZ (2,4,6‐tripyridyl‐s‐triazine), and a 20 mM solution of ferric chloride in a volume ratio of 10:1:1, respectively. The extract (25 μL) was mixed with the FRAP reagent (280 μL). The mixture was allowed to incubate for 30 min at 37°C. Absorbance was measured at 593 nm. Ascorbic acid (0–50 μg/mL) was used for standard curve creation. The results were presented as mg of AAE/g of the sample.

#### 2.3.6 ABTS Evaluation

The ABTS assay, as described by Sulastri, Zubair (Vella, Cautela, and Laratta 2019) was used with modifications. A freshly prepared ABTS^+^ dye was prepared by mixing 1.25 mL of a 7 mmol/L ABTS solution with 22 μL of 140 mmol/L potassium persulfate solutions, followed by a 16‐h incubation period in darkness at room temperature to facilitate radical formation. The ABTS reagent (290 μL) was combined with 10 μL of the sample solution. The mixture was incubated for 6 min in darkness (25°C). Absorbance was recorded at 734 nm. Ascorbic acid (0–150 μg/mL) was used for standard curve creation. The results were presented as mg AAE/g of the sample.

#### 2.3.7 Reducing Power Assay

The RPA assay was conducted according to the method reported by Ali et al. (2021). The extract (20 μL) was mixed with 20 μL of 1% potassium ferricyanide K_3_[Fe (CN)_6_] and 50 μL of 0.2 M phosphate buffer. The mixture was heated in a water bath at 25°C for 20 min. Following this, 20 μL of 10% trichloroacetic acid was added. The solution was then centrifuged at 3000 rpm for 10 min. A 50 μL portion of the supernatant was collected and mixed with distilled water (50 μL) and 0.1% FeCl_3_ (10 μL). Absorbance was measured at 750 nm. A standard curve was established using ascorbic acid concentrations ranging from 0 to 300 μg/mL, with results presented as mg AAE/g.

#### 2.3.8 Hydroxyl Radical Scavenging Activity

The ^•^OH‐RSA assay was performed according to the method reported by Smirnoff and Cumbes (1989). A mixture of 50 μL extract, 50 μL of FeSO_4_·7H_2_O (6 mM), and 50 μL of 6 mM H_2_O_2_ (30%) were added in sequence. This solution was incubated at 25°C for 10 min. Thereafter, 50 μL of 3‐hydroxybenzoic acid (6 mM) was added. Absorbance was read at 510 nm. Ascorbic acid (0–300 μg/mL) was used for a standard curve creation. The results were reported as mg AAE/g of the sample.

#### 2.3.9 Ferrous Ion Chelating Activity

The FICA value was quantified using a method adapted from Dinis, Madeira, and Almeida (1994). Initially, the extract (15 μL) was combined with distilled water (85 μL). Then, 50 μL of a 1:15 diluted 2 mM ferrous chloride solution and 50 μL of a 1:6 diluted 5 mM ferrozine solution were added to this mixture. The resulting solution was incubated at room temperature for 10 min, and the absorbance was measured at 562 nm. A standard curve was created using ethylenediaminetetraacetic acid (EDTA). The results were presented as mg EDTA equivalent/g of the sample.

#### 2.3.10 Total Antioxidant Capacity

The assessment of TAC in the samples was conducted through the modified phosphomolybdate method, as reported by Du et al. (2021). Briefly, a phosphomolybdate reagent was formulated, which consisted of sulfuric acid (0.6 M), ammonium molybdate (4 mM), and sodium phosphate (20 mM). Then, 40 μL of the extract was dispensed into 260 μL of the reagent and subjected to an incubation period of 90 min at 90°C. Absorbance was read at 695 nm. The standard curve was created using ascorbic acid (0–200 μg/mL). The results were presented as mg AAE/g of the sample.

### 2.4 LC‐ESI‐QTOF‐MS/MS Analysis

This analysis was performed according to the methodology described by Zhong et al. (2020). An HPLC system was connected to an Agilent 6520 LC‐ESI‐QTOF‐MS/MS platform (Agilent Technologies, CA, USA). Chromatographic separation was achieved using a Synergi Hydro‐RP 80 Å reverse phase column (250 × 4.6 mm; particle size = 4 μm) and a protected C18 ODS guard column. Eluent A was a mix of water and acetic acid (98:2, *v*/*v*). Eluent B was a combination of acetonitrile, water, and acetic acid (50:49.5:0.5, *v*/*v*/*v*). The elution protocol was initiated with a degassing of both mobile phases for 15 min at 21°C. The elution gradient commenced at 10% eluent B, progressively increasing to 25% at 20 min, 35% at 30 min, 40% at 40 min, then advancing to 55% at 70 min, peaking at 80% by 75 min, and 100% B from 77 to 79 min, 10% B from 82 to 85 min.

Ionization was enhanced with precisely set capillary (3.5 kV) and nozzle (500 V) voltages, nitrogen gas at 45 psi nebulizing and drying at 300°C, and sheath gas at 11 L/min and 250°C. Mass spectrometric analysis covered a broad *m/z* range (50–1300 amu) using automated MS/MS fragmentation at 10, 15, and 30 eV to identify positive and negative ion peaks. The MassHunter workstation software (Agilent Technologies, Santa Clara, CA, USA) was utilized for instrument control, data acquisition, and processing during the experiment.

### 2.5 Statistical Analysis

Each assay was replicated three times and presented as mean ± standard deviation. Data analysis was performed via Minitab 19 (Minitab for Windows Release 19, Minitab Inc., Chicago, USA) utilizing a one‐way analysis of variance (ANOVA). Tukey's HSD post hoc test was used for means comparison. The significance level was established at *p* < 0.05.

## 3 Results and Discussion

### 3.1 Polyphenol Compound Concentration

Samples sourced from QLD had generally higher TPC, TFC, and TCT values than samples from WAU (Table 1). Among them, QLD1 displayed the greatest TPC (5.97 ± 0.55 mg GAE/g), which is significantly higher than the value reported by Gharsallah et al. (2023) from 1.1 to 2.1 mg GAE/g dry weight. However, Shih et al. (2011) reported a higher value from 71.9 to 134.4 mg GAE/g. Geographical location and weather conditions between the two regions can potentially result in significant differences. For example, total polyphenol compounds were much higher in winter than in summer samples. Iqbal and Bhanger (2006) reported the same results and explained that this could be due to the degradation of polyphenols due to the increase in mono‐linear oxygen under UV irradiation. Moreover, Sulastri et al. (2018) reported that different altitudes also resulted in changes in polyphenol content in moringa. Moringa cultivated at medium altitudes exhibited higher total polyphenol, flavonoid, and quercetin contents compared to moringa plants cultivated at very low and high altitudes (15–150 m above sea level). Therefore, the different geographical locations and climates of Queensland and Western Australia may have contributed to this significant difference.

**TABLE 1:** Estimation of polyphenol compounds from moringa pods in Queensland (QLD) and Western Australia (WAU).

Samples	TPC (mg GAE/g)	TFC (mg QE/g)	TCT (mg CE/g)
QLD1	5.97 ± 0.55^a^	8.88 ± 0.49^a^	1.95 ± 0.10^c^
QLD2	5.00 ± 0.15^b,c^	4.84 ± 0.08^c^	2.07 ± 0.06^bc^
QLD3	4.58 ± 0.37^b,cd^	3.98 ± 0.13^def^	2.65 ± 0.14^a^
QLD4	3.46 ± 0.15^fg^	4.44 ± 0.09^cd^	2.34 ± 0.14^ab^
QLD5	2.32 ± 0.09^h^	4.44 ± 0.15^cd^	2.38 ± 0.15^ab^
QLD6	1.72 ± 0.10^h^	2.57 ± 0.10^h^	1.57 ± 0.04^d^
QLD7	1.64 ± 0.07^h^	3.41 ± 0.09^efg^	1.55 ± 0.09^d^
QLD average	3.53 ± 0.21	4.62 ± 0.16	2.07 ± 0.10
WAU5	2.84 ± 0.13^g^	7.98 ± 0.59^b^	2.15 ± 0.20^bc^
WAU7	3.91 ± 0.27^def^	4.31 ± 0.05^cd^	1.51 ± 0.12^de^
WAU10	3.48 ± 0.24^g^	3.27 ± 0.18^de^	1.42 ± 0.14^de^
WAU15	3.07 ± 0.27^cde^	4.08 ± 0.28^h^	1.49 ± 0.03^c^
WAU17	4.31 ± 0.25^fg^	2.68 ± 0.14^fgh^	1.92 ± 0.07^de^
WAU20	3.48 ± 0.24^b^	3.27 ± 0.18^gh^	1.42 ± 0.14^e^
WAU32	3.14 ± 0.32^efg^	3.13 ± 0.05^cd^	1.19 ± 0.09^d^
WAU average	3.48 ± 0.26	4.24 ± 0.20	1.60 ± 0.10

*Note:* Values are mean of 3 replications ± standard deviation. ^a‐h^Means within the same column with dissimilar superscript letters differ (*p* < 0.05).

The TFC value ranged from 2.57 to 8.88 mg QE/g in QLD samples and 2.68 to 7.98 mg QE/g in WAU samples. The highest TPC content was quantified in QLD1 at 8.88 mg GAE/g, which agrees with the findings of Braham et al. (2020), reporting that the TPC value ranged from 3.7 to 9.1 mg QUE/g depending on the extraction solvent. The authors also demonstrated that flavonoid content in moringa was also strongly influenced by the extraction solvent, explaining the large difference between the results of this experiment and those reported in the literature. Sulastri et al. (2018) reported the influence of agroclimatic conditions on TFC and TPC in moringa leaves sourced from various regions and noted a correlation between the geographical location and the TFC, similar to the trend observed for TPC. The authors suggested that this correlation might stem from the polyphenol properties of flavonoids. Hani et al. (2017) also highlighted the importance of factors such as maturity stage, climate, post‐harvest handling, and solvent type on TPC and TFC measurements. In support, our current experiment demonstrated consistently elevated levels of both TPC and TFC in the samples obtained from Queensland.

The TCT analysis showed that the samples originating from QLD had significantly higher values than those originating from WAU. On average, the QLD samples had a value of 2.07 ± 0.10 mg CE/g versus an average of 1.60 ± 0.10 mg CE/g in WAU samples. Additionally, the individual samples from QLD mostly had values that exceeded those of all the WAU samples. The average tannins level in this experiment was significantly lower than previously reported which reported at 4.9 mg catechin/g (Adisakwattana and Chanathong 2011). Tannins possess anticancer, anti‐inflammatory, and anti‐hepatotoxic properties (Vergara‐Jimenez, Almatrafi, and Fernandez 2017). However, consuming tannins in high doses can be toxic and may lead to adverse side effects such as abdominal pain, vomiting, nausea, and liver damage (Baldwin and Booth 2022). Therefore, the moderate tannin content of moringa pods makes them more suitable for use in the food industry.

### 3.2 Antioxidant Activity

The DPPH and ABTS assay results consistently showed that the samples from QLD had higher mean values (Table 2). The DPPH and ABTS values for QLD samples averaged 2.87 and 15.0 mg AAE/g, respectively. Specifically, the highest antioxidant activity, as determined by the DPPH and ABTS tests, was quantified in QLD6 and QLD5 samples, respectively. Conversely, the mean values of DPPH and ABTS for WAU samples were 2.74 and 12.9 AAE/g, respectively. A review of the existing literature reveals discrepancies in the antioxidant activities observed in the current experiment. For instance, Nobossé, Fombang, and Mbofung (2018) documented ABTS values ranging from 3.44 to 3.86 mg AAE/g in moringa samples. These variations in results are likely attributed to factors such as the tree's age and the solvents used for extraction.

**TABLE 2:** Estimation of antioxidant capacity of moringa pods in Queensland (QLD) and Western Australia (WAU).

Samples	DPPH (mg AAE/g)	ABTS (mg AAE/g)	FRAP (mg AAE/g)	RPA (mg AAE/g)	•OH‐RSA (mg AAE/g)	FICA (mg EDTA/g)	TAC (mg AAE/g)
QLD1	2.65 ± 0.14^cde^	14.8 ± 0.8^cde^	14.6 ± 1.4^bcd^	3.50 ± 0.09^e^	96.3 ± 3.1^bc^	0.80 ± 0.07^bcd^	2.48 ± 0.16^b^
QLD2	2.93 ± 0.12^bc^	15.1 ± 0.5^bcd^	14.5 ± 0.8^bcd^	5.82 ± 0.578^d^	88.1 ± 2.4^cd^	0.73 ± 0.04^cd^	2.37 ± 0.13^b^
QLD3	2.84 ± 0.14^bcd^	16.0 ± 0.1^bc^	13.5 ± 0.5^cde^	3.04 ± 0.15^ef^	97.2 ± 3.4^bc^	0.79 ± 0.06^bcd^	2.96 ± 0.10^a^
QLD4	2.76 ± 0.19^bcde^	16.9 ± 0.9^ab^	12.7 ± 0.5^de^	7.85 ± 0.26^c^	84.5 ± 5.4^d^	0.83 ± 0.01^bcd^	2.55 ± 0.21^b^
QLD5	3.05 ± 0.16^bc^	17.5 ± 1.5^a^	14.7 ± 0.6^bcd^	5.57 ± 0.16^d^	95.8 ± 5.0^bc^	0.83 ± 0.01^bcd^	3.11 ± 0.24^a^
QLD6	3.23 ± 0.21^ab^	12.4 ± 0.6^fg^	9.30 ± 0.2^f^	2.31 ± 0.20^f^	100.6 ± 1.4^b^	1.05 ± 0.08^a^	0.44 ± 0.03^e^
QLD7	2.63 ± 0.14^cde^	12.4 ± 0.7^fg^	11.6 ± 0.4^ef^	2.94 ± 0.06^ef^	98.6 ± 4.0^b^	0.84 ± 0.02^bc^	2.44 ± 0.13^b^
QLD average	2.87 ± 0.16	15.0 ± 0.74	13.0 ± 0.64	4.43 ± 0.21	94.4 ± 3.52	0.84 ± 0.04	2.34 ± 0.14
WAU5	3.61 ± 0.11^a^	13.6 ± 0.9^def^	15.1 ± 0.7^bc^	3.30 ± 0.16^e^	95.7 ± 3.01^bc^	1.06 ± 0.08^a^	0.27 ± 0.01^e^
WAU7	2.36 ± 0.22^de^	14.6 ± 0.2^cde^	15.7 ± 1.1^bc^	0.74 ± 0.07^g^	119.1 ± 5.8^a^	0.87 ± 0.01^bc^	1.84 ± 0.03^c^
WAU10	2.33 ± 0.04^e^	12.1 ± 0.1^efg^	13.9 ± 0.2^bcd^	7.81 ± 0.12^a^	124.8 ± 1.5^a^	0.91 ± 0.08^bc^	1.71 ± 0.07^b^
WAU15	2.32 ± 0.18^bc^	13.0 ± 0.5^g^	14.6 ± 0.4^a^	12.34 ± 0.23^f^	124.6 ± 1.6^a^	0.84 ± 0.07^bcd^	1.31 ± 0.01^d^
WAU17	3.03 ± 0.20^de^	11.4 ± 0.7^fg^	18.4 ± 0.9^cd^	2.44 ± 0.08^c^	117.3 ± 1.0^a^	0.80 ± 0.04^ab^	1.82 ± 0.09^c^
WAU20	2.33 ± 0.04^cde^	12.9 ± 0.1^fg^	13.8 ± 0.2^ab^	7.81 ± 0.12^b^	124.8 ± 1.5^a^	0.91 ± 0.08^d^	1.71 ± 0.07^c^
WAU32	2.83 ± 0.19^a^	12.3 ± 0.4^defg^	16.3 ± 1.3^ab^	10.2 ± 0.50^c^	122.9 ± 2.4^a^	0.68 ± 0.05^bcd^	1.74 ± 0.14^cd^
WAU average	2.74 ± 0.17	12.9 ± 0.4	15.8 ± 0.8	3.50 ± 0.09^e^	118.5 ± 2.6	0.84 ± 0.05	1.46 ± 0.07

*Note:* Values are mean of 3 replications ± standard deviation. ^a–g^Means within the same column with dissimilar superscript letters differ (*p* < 0.05).

In contrast to the DPPH and ABTS assay results, the FRAP assay data indicated higher values for the samples from WAU as compared to those from QLD (15.8 ± 0.8 vs. 13.0 ± 0.64 mg AAE/g), with WAU17 exhibiting the maximum reducing power (18.4 mg AAE/g), while the lowest was observed in QLD6 (9.30 mg AAE/g). This variation may be attributed to differences in antioxidant assay procedures. The FRAP assay evaluates antioxidant ability based on the reducing power, which involves the ability of antioxidant chemicals to donate electrons and reduce Fe^3+^ to Fe^2+^ ions.

The RPA assay utilizes a colorimetric method where the reduction of the Fe^3+^/ferricyanide complex induces a color change upon conversion to the ferrous form. In this experiment, the RPA results mirrored the trend observed in the FRAP assay, showing generally higher values for the samples from WAU. Among these samples, WAU15 exhibited the highest reducing power (12.3 ± 0.23 mg AAE/g). The consistent findings from both assays support the reliability of the results and confirm the stronger reducing power of the samples originating from WAU.

The WAU samples had generally higher ^•^OH‐RSA activity than the QLD samples (118.5 vs. 94.4 mg AAE/g). Several investigations have stated that flavonoids are effective scavengers of hydroxyl free radicals, implying the contribution of flavonoids to the scavenging activity (Hu et al. 2021). However, this study found no statistically significant association between TFC and ^•^OH‐RSA. The ^•^OH‐RSA assay quantifies the ability to scavenge the highly reactive hydroxyl radical (^•^OH) generated in the Fenton reaction. Chronic exposure to this radical may cause significant health concerns.

Polyphenol compounds can bind to metal ions like Fe^2+^, providing a means to assess their antioxidant capabilities. Both samples from QLD and WAU demonstrated significant metal chelating capabilities, likely attributed to their higher flavonoid content as flavonoids are known to be linked with their chelating capacity. The assay results showed a range of 0.73–1.06 mg EDTA/g, averaging 0.84 mg EDTA/g across all samples.

Polyphenolic compounds, including flavonoids and phenolic acids, may contribute to antioxidant ability through following mechanisms: (1) neutralization of free radicals, such as reactive oxygen species (ROS) and reactive nitrogen species (RNS), by donating hydrogen atoms or electrons to reduce oxidative stress; (2) inhibition of enzymes involved in the production of these radicals or chelating trace metals that catalyze ROS/RNS formation; and (3) regulating or enhancing endogenous antioxidant defense systems (Hassan et al. 2021). Polyphenols such as quercetin and caffeic acid determined by LC‐ESI‐QTOF‐MS in this study demonstrated strong free radical scavenging and metal‐chelating abilities, which are likely responsible for the observed antioxidant effects. These mechanisms may explain the higher antioxidant activities in the QLD samples as indicated by DPPH and ABTS assays. These findings are consistent with previous findings on how environmental factors, such as climate, soil, and UV radiation, on the synthesis and accumulation of bioactive compounds in plants (Özcan 2020).

### 3.3 Correlation Between Polyphenol Content and Antioxidant Assays

The TCT strongly correlated with both ABTS and ^•^OH‐RSA antioxidant assays (Table 3). Specifically, a correlation coefficient (*r*) of 0.765 for TCT and ABTS indicates a substantial positive relationship, and the high correlation suggests that the condensed tannins present in moringa pods are potent radical scavengers. This is possibly owing to their polyphenol structures which can donate hydrogen atoms to neutralize free radicals. Similarly, the strong positive correlation of TCT with ^•^OH‐RSA (*r* = 0.744) may indicate the effectiveness of condensed tannins in neutralizing hydroxyl radicals. The ability of moringa pod to counteract these radicals highlights its potential application as a potent natural antioxidant source within the food industry.

**TABLE 3:** Pearson's correlation coefficients.

Variables	TPC	TFC	TCT	DPPH	FRAP	ABTS	^•^OH‐RSA	RPA	FICA
TFC	0.319								
TCT	0.015	0.346							
DPPH	0.216	0.229	0.438						
FRAP	0.495	0.124	0.062	0.074					
ABTS	0.001	0.345	0.765**	0.073	0.136				
^•^OH‐RSA	0.105	0.399	0.744**	0.502	0.482	0.650*			
RPA	0.085	0.156	0.297	0.360	0.145	0.061	0.362		
FICA	0.594*	0.132	0.027	0.415	0.499	0.153	0.158	0.384	
TAC	0.209	0.023	0.472	0.337	0.080	0.630*	0.333	0.014	0.661**

* Indicates a significant correlation with *p* ≤ 0.05.

** Indicates a significant correlation with *p* ≤ 0.01.

A moderate positive correlation (*r* = 0.594) as shown in FICA and TPC may indicate that polyphenol content is significantly related to iron‐chelating activity. Polyphenol compounds play a role in chelating ferric ions, significantly reducing oxidative stress. A robust correlation between TAC and ABTS (*r* = 0.630) suggests that samples with higher total antioxidant capacities exhibit higher scavenging activity against the ABTS radical cation, potentially validating the consistency between these assays in measuring the antioxidant potential.

Previous studies have reported a positive correlation between polyphenol content, suggesting that as the content of polyphenol compounds increases, the overall antioxidant capacity also increases (González‐Romero et al. 2020). This observation aligns with the findings of our study, where higher TAC values were observed in QLD samples along with elevated levels of TPC, TFC, and TCT.

**FIGURE 1:**
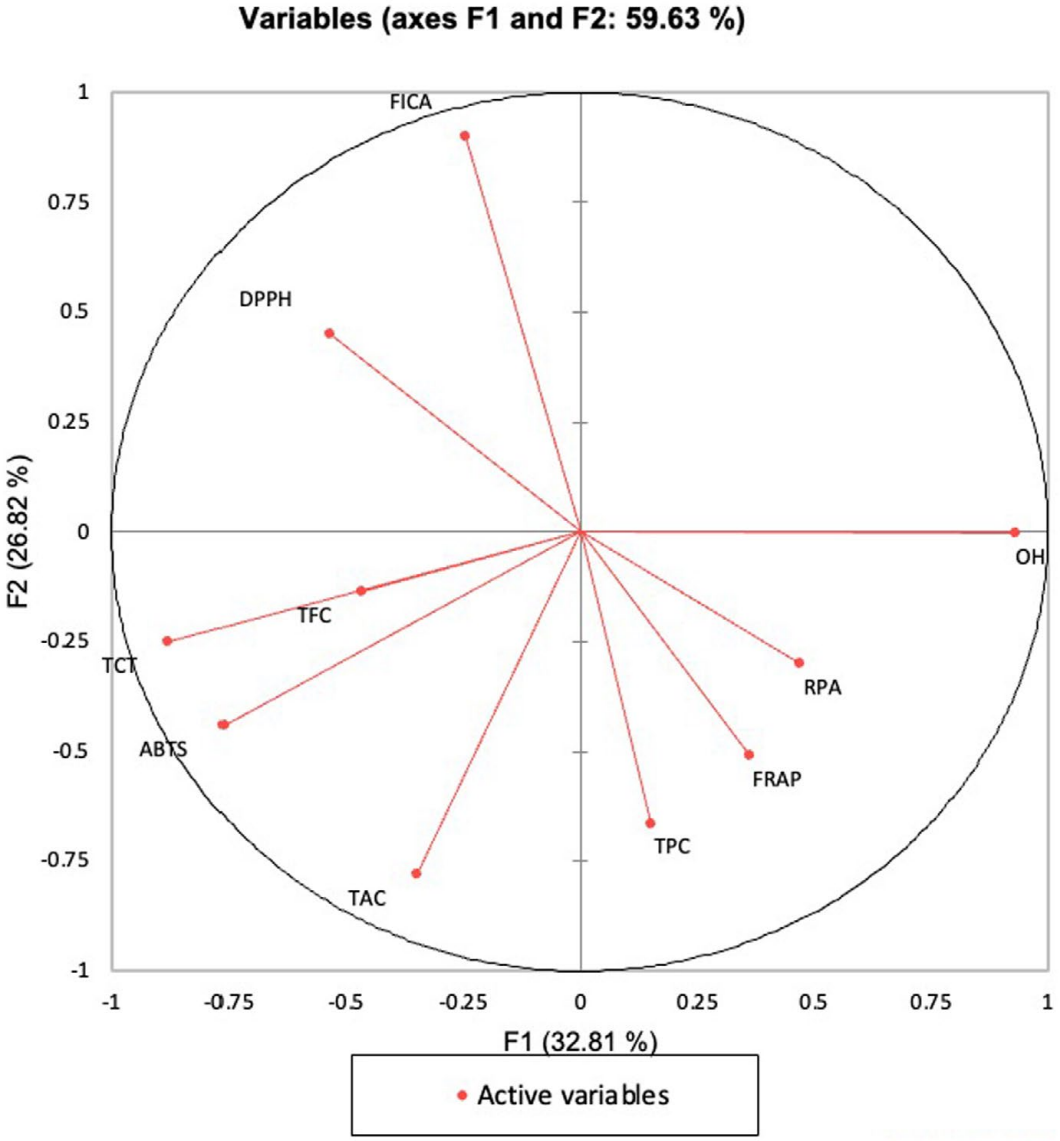
Principal component analysis of antioxidant components and polyphenol compounds.

Figure 1 illustrates the Principal Component Analysis (PCA) of antioxidant components, including TPC, TFC, TCT, and individual antioxidant activity assays such as DPPH, ABTS, FRAP, ^•^OH‐RSA in the moringa pod samples originated from QLD and WAU. The ABTS, TFC, and TCT vectors were closely positioned in the loading plot, indicating a high correlation between these antioxidant compounds. These are concentrated on the negative side of principal component 1 (F1), accounting for 32.81% of the variance in the data. The antioxidant assays form a distinct cluster, reflecting their cumulative contribution to total antioxidant activity but independence from phenolic acid and flavonoid concentration.

### 3.4 Polyphenol Characterization

Identification of polyphenol components in the moringa pod samples from two regions was performed using qualitative analysis by LC‐ESI‐QTOF‐MS. Results with a mass inaccuracy exceeding ± 5 ppm were excluded. In general, there was a considerable diversity of antioxidant compounds within the samples from both regions. In total, 111 polyphenol compounds were identified, comprising 32 phenolic acids, 54 flavonoids, 13 other phenolic compounds, 3 lignans, and 9 stilbenes (Table 4).

**TABLE 4:** Characterization of polyphenol compounds in moringa pod by LC‐ESI‐QTOF MS/MS.

No.	Proposed compounds	Molecular formula	RT (min)	Ionization (ESI + /ESI−)	Molecular weight	Theoretical (*m/z*)	Observed (*m/z*)	MS^2^ Productions	Error (ppm)	Moringa samples
Phenolic acids										
Hydroxybenzoic acids										
1	Protocatechuic acid	C_7_H_6_O_4_	8.601	[M + H]^+^	154.0271	155.0344	155.0338	109, 139	−3.9	^a^QLD, WAU5
2	4‐Hydroxybenzoic acid 4‐*O*‐glucoside	C_13_H_16_O_8_	35.595	[M − H]^−^	300.0867	299.0794	299.0795	255, 137	0.3	^a^QLD1, QLD5
3	Gallic acid	C_7_H_6_O_5_	37.239	[M − H]^−^	170.0231	169.0158	169.0157	125	−0.6	^a^QLD1, QLD7
4	Paeoniflorin	C_23_H_28_O_11_	54.258	[M − H]^−^	480.1648	479.1575	479.1562	449, 327	−2.7	^a^QLD1, QLD2, QLD5, WAU15, WAU20
5	4‐*O*‐Methylgallic acid	C_8_H_8_O_5_	58.44	[M + H]^+^	184.0388	185.0461	185.0461	124, 170	0.0	^a^WAU5, WAU20, WAU 32
6	Benzoic acid	C_7_H_6_O_2_	60.026	^b^[M − H]^−^	122.0375	121.0302	121.0302	77	0.0	^a^QLD1, QLD 2, QLD 3, QLD5, QLD 6, QLD7, WAU5, WAU7, WAU10 WAU15, WAU32
7	Ellagic acid	C_14_H_6_O_8_	61.909	[M − H]^−^	302.0075	301.0002	301.0007	257	1.7	QLD4
8	Protocatechuic acid 4‐*O‐*glucoside	C_13_H_16_O_9_	65.464	[M + H]^+^	316.079	317.0863	317.0856	153	−2.2	^a^QLD2, QLD6, WAU7
9	3,4‐*O*‐Dimethylgallic acid	C_9_H_10_O_5_	67.076	[M + H]^+^	198.0546	199.0619	199.0619	153, 139, 125, 111	0.0	^a^QLD6, QLD7, WAU20
10	2‐Hydroxybenzoic acid	C_7_H_6_O_3_	68.424	^b^[M − H]^−^	138.0322	137.0249	137.0249	93, 65	0.0	^a^QLD1, QLD2, QLD4, QLD5, QLD6, QLD7, WAU10, WAU 7, WAU15
Hydroxycinnamic acids										
11	Caffeoyl glucose	C_15_H_18_O_9_	11.187	^b^[M − H]^−^	342.0966	341.0893	341.0891	179, 161	−0.6	^a^QLD1, QLD3, QLD5, QLD 6, QLD7, WAU5 WAU10, WAU15, WAU20, WAU32
12	1‐Sinapoyl‐2‐feruloylgentiobiose	C_33_H_40_O_18_	11.702	^b^[M − H]^−^	724.2205	723.2132	723.2124		−1.1	^a^QLD7, WAU5, WAU10, WAU17
13	4,5‐Dicaffeoylquinic acid	C_25_H_24_O_12_	11.828	[M − H]^−^	516.1305	515.1232	515.1232	191	0.0	^a^QLD3, QLD6, QLD7, WAU5, WAU7, WAU10, WAU15, WAU17, WAU20, WAU32
14	Ferulic acid 4‐*O*‐glucuronide	C_16_H_18_O_10_	13.618	^b^[M − H]^−^	370.0899	369.0826	369.0825	193, 175	−0.3	^a^QLD4, QLD5, WAU20
15	Rosmarinic acid	C_18_H_16_O_8_	28.355	[M − H]^−^	360.086	359.0787	359.0785	197, 179	−0.6	^a^QLD1, QLD 4
16	Caffeoyl C1‐glucuronide	C_15_H_16_O_10_	39.359	^b^[M − H]^−^	356.0742	355.0669	355.0663		−1.7	^a^QLD1, QLD4, QLD6, WAU7, WAU10, WAU32
17	Ferulic acid 4‐*O*‐glucoside	C_16_H_20_O_9_	41.558	[M − H]^−^	356.1126	355.1053	355.1045	193, 178, 149, 134	−2.3	^a^QLD1, QLD3, QLD5, QLD7, WAU5, WAU10, WAU15, WAU17, WAU20
18	Caffeic acid	C_9_H_8_O_4_	41.923	[M − H]^−^	180.0438	179.0365	179.0368	143, 135	1.7	QLD1
19	Ferulic acid	C_10_H_10_O_4_	48.519	[M − H]^−^	194.0589	193.0516	193.0519	178, 149	1.6	^a^QLD1, QLD3, QLD5
20	m‐Coumaric acid	C_9_H_8_O_3_	50.67	[M − H]^−^	164.0462	163.0389	163.0388	148, 119	−0.6	^a^QLD1, QLD6, WAU17
21	3‐Sinapoylquinic acid	C_18_H_22_O_10_	52.062	^b^[M − H]^−^	398.1217	397.1144	397.1144	233, 179	0.0	^a^QLD1, QLD2, QLD3, QLD6, QLD7, WAU5, WAU7, WAU10, WAU15, WAU17, WAU 20, WAU32
22	Feruloyl tartaric acid	C_14_H_14_O_9_	52.071	[M − H]^−^	326.063	325.0557	325.0551	193, 149	−1.8	^a^WAU5, WAU7, WAU10, WAU15, WAU17, WAU32, QLD2, QLD3, QLD 5, QLD6, QLD7
23	p‐Coumaroyl tartaric acid	C_13_H_12_O_8_	52.55	[M − H]^−^	296.0505	295.0432	295.0423	115	−3.1	WAU10
24	5‐p‐Coumaroylquinic acid	C_16_H_18_O_8_	63.523	^b^[M − H]^−^	338.103	337.0957	337.0956	163, 191	−0.3	^a^QLD1, QLD2, QLD3, QLD4, QLD5, QLD6, QLD7, WAU5 WAU7, WAU10, WAU15, WAU32
25	5‐Feruloylquinic acid	C_17_H_20_O_9_	65.315	^b^[M + H]^+^	368.1076	369.1149	369.1147	173, 191	−0.5	^a^QLD1, QLD2, QLD3, QLD4, QLD5, QLD6, QLD7, WAU7, WAU 10WAU15, WAU17, WAU32
26	p‐Coumaric acid 4‐*O*‐glucoside	C_15_H_18_O_8_	66.484	^b^[M + H]^+^	326.1017	327.109	327.1087	163	−0.9	^a^QLD1, QLD3, WAU5, WAU 32
27	Cinnamoyl glucose	C_15_H_18_O_7_	66.526	^b^[M + H]^+^	310.1037	311.111	311.111	147, 131, 103	0.0	^a^QLD3, WAU15, WAU32
28	3‐Caffeoylquinic acid	C_16_H_18_O_9_	67.638	^b^[M + H]^+^	354.0935	355.1008	355.1005	253, 190, 144	−0.8	^a^QLD1, QLD4, WAU7, WAU15, WAU17, WAU20
29	p‐Coumaroyl malic acid	C_13_H_12_O_7_	68.652	^b^[M + H]^+^	280.0568	281.0641	281.0641	163, 119	0.0	^a^WAU10, WAU15
Hydroxyphenylacetic acids										
30	3,4‐Dihydroxyphenylacetic acid	C_8_H_8_O_4_	42.768	[M − H]^−^	168.0438	167.0365	167.0362	149, 123	−1.8	^a^QLD1, QLD6, QLD7
Hydroxyphenylpropanoic acids										
31	Dihydrocaffeic acid 3‐*O*‐glucuronide	C_15_H_18_O_10_	9.451	[M − H]^−^	358.0935	357.0862	357.0861	181	−0.3	^a^QLD1, QLD2, QLD4, QLD5, QLD6, QLD7
32	Dihydroferuloylglycine	C_12_H_15_NO_5_	31.054	^b^[M − H]^−^	253.0934	252.0861	252.086	173,151	−0.4	^a^QLD6, WAU32
Flavonoids										
Anthocyanins										
33	Pelargonidin	C_15_H_11_O_5_	12.015	[M − H]^−^	271.0611	270.0538	270.0534	225, 215	−1.5	QLD5
34	Cyanidin 3‐*O*‐(6″‐acetyl‐glucoside)	C_23_H_23_O_12_	63.96	[M − H]^−^	491.1195	490.1122	490.1118		−0.8	^a^QLD1, QLD2, QLD3, QLD5, QLD6 QLD7, WAU7
35	Petunidin 3,5‐*O*‐diglucoside	C_28_H_33_O_17_	68.834	[M + H]^+^	641.1706	642.1779	642.1797		2.8	QLD2
Dihydrochalcones										
36	Dihydromyricetin 3‐*O*‐rhamnoside	C_21_H_22_O_12_	63.353	[M − H]^−^	466.1111	465.1038	465.1037	301	−0.2	^a^QLD1, WAU5, WAU32
37	Dihydroquercetin 3‐*O*‐rhamnoside	C_21_H_22_O_11_	63.86	[M − H]^−^	450.1138	449.1065	449.1051		−3.1	WAU17
38	3‐Hydroxyphloretin 2′‐*O*‐glucoside	C_21_H_24_O_11_	66.534	[M − H]^−^	452.1313	451.124	451.1235	289, 273	−1.1	^a^QLD3, QLD4, QLD5, QLD7, WAU7
Flavanols										
39	4″‐*O*‐Methylepigallocatechin 3‐*O*‐gallate	C_23_H_20_O_11_	9.811	[M − H]^−^	472.1023	471.095	471.093	169, 319	−4.2	QLD4
40	Prodelphinidin dimer B3	C_30_H_26_O_14_	11.185	[M + H]^+^	610.1344	611.1417	611.1444	469, 311, 291	4.4	QLD1
41	(+)‐Gallocatechin 3‐*O*‐gallate	C_22_H_18_O_11_	11.728	[M − H]^−^	458.0865	457.0792	457.079	289, 169, 125	−0.4	QLD5
42	4′‐*O*‐Methyl‐(−)‐epigallocatechin 7‐*O*‐glucuronide	C_22_H_24_O_13_	12.905	[M − H]^−^	496.1218	495.1145	495.1145	451, 313	0.0	QLD6
43	(+)‐Gallocatechin	C_15_H_14_O_7_	60.666	^b^[M + H]^−^	306.0736	305.0663	305.0657		−2.0	^a^QLD1, QLD6, QLD7, WAU5, WAU7, WAU10, WAU20, WAU32
44	Theaflavin	C_29_H_24_O_12_	65	^b^[M + H]^+^	564.1295	565.1368	565.1368		0.0	^a^WAU5, WAU7 WAU10, WAU15 WAU17, WAU20 WAU32, QLD4, QLD5, QLD6, QLD7
45	Procyanidin dimer B7	C_30_H_26_O_12_	65.848	^b^[M − H]^−^	578.1434	577.1361	577.1353		−1.4	^a^QLD1, QLD7, WAU5, WAU7, WAU10, WAU15, WAU20
Flavanones										
46	Sakuranetin	C_16_H_14_O_5_	13.922	[M + H]^+^	286.085	287.0923	287.0923	255	0.0	WAU5
47	Hesperetin 3ʹ‐*O*‐glucuronide	C_22_H_22_O_12_	63.943	^b^[M − H]^−^	478.1135	477.1062	477.1054	301, 175, 113, 85	−1.7	^a^QLD1, QLD2, QLD3, QLD5, QLD6, QLD7, WAU5, WAU7, WAU10, WAU15, WAU20
48	Naringin 4ʹ‐*O*‐glucoside	C_33_H_42_O_19_	64.552	^b^[M − H]^−^	742.2303	741.223	741.2223	433, 271	−0.9	^a^QLD3, QLD5, WAU5, WAU10
49	Isoxanthohumol	C_21_H_22_O_5_	64.943	^b^[M + H]^+^	354.1471	355.1544	355.1529		−4.2	^a^QLD2, QLD7, WAU7, WAU10, WAU15, WAU17
50	Narirutin	C_27_H_32_O_14_	64.996	^b^[M + H]^+^	580.1777	581.185	581.1834	271	−2.8	^a^QLD4, QLD6, WAU5, WAU7, WAU10, WAU15, WAU17, WAU20
51	8‐Prenylnaringenin	C_20_H_20_O_5_	65.491	[M + H]^+^	340.1311	341.1384	341.1383	285	−0.3	^a^QLD3, QLD5, QLD6, QLD7
52	Neohesperidin	C_28_H_34_O_15_	68.158	^b^[M + H]^+^	610.1897	611.197	611.194		−4.9	WAU5, WAU7, WAU10, WAU15, WAU17, WAU20, WAU32
Flavones										
53	Nepetin	C_16_H_12_O_7_	3.082	^b^[M + H]^+^	316.0561	317.0634	317.0624		−3.2	^a^QLD2, QLD5, WAU5, WAU7, WAU10, WAU15, WAU17, WAU20, WAU32
54	Luteolin 7‐*O*‐(2‐apiosyl‐glucoside)	C_26_H_28_O_15_	9.172	[M + H]^+^	580.1392	581.1465	581.146		−0.9	QLD4
55	Apigenin 6,8‐di‐*C*‐glucoside	C_27_H_30_O_15_	11.409	[M − H]^−^	594.164	593.1567	593.157	503, 473	0.5	^a^WAU10, WAU15, WAU20, WAU 32
56	Nobiletin	C_21_H_22_O_8_	14.049	^b^[M − H]^−^	402.1305	401.1232	401.1221		−2.7	^a^QLD1, QLD4, WAU5, WAU7, WAU10, WAU15, WAU32
57	Apigenin 6‐*C‐*glucoside	C_21_H_20_O_10_	51.706	[M − H]^−^	432.1066	431.0993	431.0974	413, 341, 311	−4.4	QLD5
58	Neodiosmin	C_28_H_32_O_15_	51.756	^b^[M − H]^−^	608.1728	607.1655	607.1626		−4.8	^a^QLD2, QLD4, QLD5, WAU7, WAU 32
59	Gardenin B	C_19_H_18_O_7_	52.095	^b^[M − H]^−^	358.1052	357.0979	357.0965	344, 329, 311	−3.9	^a^QLD1, QLD2, QLD 5, WAU10, WAU15, WAU17, WAU20, WAU32
60	Isorhoifolin	C_27_H_30_O_14_	55.222	^b^[M − H]^−^	578.1689	577.1616	577.1588		−4.9	^a^WAU5, WAU7, WAU15, WAU20, WAU32
61	Apigenin 7‐*O*‐apiosyl‐glucoside	C_26_H_28_O_14_	63.862	[M + H]^+^	564.145	565.1523	565.152		−0.5	QLD1
62	Apigenin 7‐*O*‐diglucuronide	C_27_H_26_O_17_	64.477	[M − H]^−^	622.1174	621.1101	621.1105		0.6	^a^QLD4, QLD5
63	Scutellarein	C_15_H_10_O_6_	64.981	^b^[M + H]^+^	286.0479	287.0552	287.0554		0.7	^a^QLD1, QLD2, QLD3, QLD5, QLD6, QLD7, WAU5, WAU7, WAU10, WAU15, WAU17, WAU20, WAU 32
Flavonols										
64	Myricetin 3‐*O*‐arabinoside	C_20_H_18_O_12_	12.27	[M − H]^−^	450.0769	449.0696	449.0707	317	2.4	WAU20
65	Kaempferol 7‐*O*‐glucoside	C_21_H_19_O_11_	50.987	[M − H]^−^	447.0948	446.0875	446.0869	162	−1.3	^a^WAU5, WAU17, WAU 20
66	Quercetin 3ʹ‐*O*‐glucuronide	C_21_H_18_O_13_	60.34	^b^[M + H]^+^	478.0767	479.084	479.0838	301	−0.4	^a^QLD4, WA15
67	Spinacetin 3‐*O*‐(2″″‐p‐coumaroylglucosyl) (1‐ > 6)‐ [apiosyl (1‐ > 2)]‐glucoside	C_43_H_48_O_24_	61.84	^b^[M + H]^+^	948.2526	949.2599	949.2594		−0.5	^a^WAU5, WAU15, WAU32
68	Myricetin 3‐*O*‐galactoside	C_21_H_20_O_13_	63.032	^b^[M − H]^−^	480.0871	479.0798	479.0802	317	0.8	^a^QLD3, QLD4, WAU 5, WAU7, WAU15, WAU17
69	Quercetin 3‐*O*‐glucosyl‐xyloside	C_26_H_28_O_16_	63.487	^b^[M − H]^−^	596.1381	595.1308	595.1293	265, 138, 116	−2.5	^a^QLD4, WAU5, WAU10
70	Quercetin 3‐*O*‐rhamnoside	C_21_H_20_O_11_	64.808	^b^[M + H]^+^	448.1011	449.1084	449.1085		0.2	^a^WAU5, WAU7, WAU17, WAU20
71	Quercetin 3‐*O*‐xylosyl‐glucuronide	C_26_H_26_O_17_	64.86	[M + H]^+^	610.1192	611.1265	611.1256	479, 303, 285, 239	−1.5	^a^QLD2, QLD4, WAU10
72	Quercetin 3‐*O*‐rutinoside	C_27_H_30_O_16_	65.399	[M − H]^−^	610.1524	609.1451	609.1437		−2.3	^a^QLD7, WAU17
73	Patuletin 3‐*O*‐glucosyl‐(1‐ > 6)‐ [apiosyl (1‐ > 2)]‐glucoside	C_33_H_40_O_22_	65.472	[M − H]^−^	788.2056	787.1983	787.1983	625, 463, 301, 271	0.0	^a^QLD2, QLD3
74	Quercetin 3‐*O*‐(6″″‐malonyl‐glucoside)	C_24_H_22_O_15_	67.477	[M − H]^−^	550.0988	549.0915	549.0915		0.0	QLD3, WAU5, WAU10, WAU20
Isoflavonoids										
75	Tectoridin	C_22_H_22_O_11_	9.017	^b^[M + H]^+^	462.114	463.1213	463.1216		0.6	^a^QLD1, QLD3, QLD7, WAU5, WAU7, WAU10
76	Dalbergin	C_16_H_12_O_4_	11.335	[M − H]^−^	268.0742	267.0669	267.0667	252, 224, 180	−0.7	WAU5
77	Formononetin 7‐*O*‐glucuronide	C_22_H_20_O_10_	11.731	[M − H]^−^	444.1082	443.1009	443.1015	267, 252	1.4	^a^WAU17, WAU20
78	Violanone	C_17_H_16_O_6_	41.17	^b^[M − H]^−^	316.0967	315.0894	315.0899	300, 285, 135	1.6	^a^QLD1, QLD2, QLD5, QLD6, WAU10, WAU17, WAU32
79	2′‐Hydroxyformononetin	C_16_H_12_O_5_	42.303	^b^[M − H]^−^	286.0829	285.0756	285.076		1.4	^a^QLD1, QLD2, QLD5, WAU15, WAU20, WAU32
80	3′‐*O*‐Methylviolanone	C_18_H_18_O_6_	49.183	^b^[M − H]^−^	330.1096	329.1023	329.1023		0.0	^a^QLD2, QLD7, WAU10, WAU15, WAU20, WAU32
81	6″‐*O*‐Acetylgenistin	C_23_H_22_O_11_	50.88	^b^[M − H]^−^	474.1184	473.1111	473.1115		0.8	^a^QLD1, QLD2, QLD3, WAU7, WAU10, WAU20
82	Prunetin	C_16_H_12_O_5_	51.938	^b^[M − H]^−^	284.0671	283.0598	283.0597		−0.4	^a^QLD2, WAU7, WAU20
83	6″‐*O*‐Malonylgenistin	C_24_H_22_O_13_	58.952	^b^[M + H]^+^	518.1058	519.1131	519.1133	271	0.4	^a^QLD3, WAU15, WAU17, WAU20
84	Glycitin	C_22_H_22_O_10_	63.998	^b^[M − H]^−^	446.1226	445.1153	445.114	285	−2.9	^a^QLD5, WAU20, WAU32
85	5,6,7,3′,4′‐Pentahydroxyisoflavone	C_15_H_10_O_7_	68.651	^b^[M − H]^−^	302.0432	301.0359	301.0357	285, 257	−0.7	^a^QLD1, QLD2, QLD3, QLD4, QLD5, QLD6, QLD7, WAU5, WAU7, WAU10, WAU15, WAU17, WAU20, WAU32
86	2‐Dehydro‐*O*‐desmethylangolensin	C_15_H_12_O_4_	68.901	[M − H]^−^	256.0756	255.0683	255.0686	135, 119	1.2	^a^QLD3, QLD4, QLD6, WAU15, WAU17, WAU20, WAU32
Other polyphenols										
Alkylphenols										
89	4‐Vinylphenol	C_8_H_8_O	9.489	^b^[M − H]^−^	120.0576	119.0503	119.0504		0.8	^a^QLD2, QLD6, QLD7, WAU5, WAU7, WAU10, WAU15, WAU 32
Hydroxybenzoketones										
87	2‐Hydroxy‐4‐methoxyacetophenone 5‐sulfate	C_9_H_10_O_7_S	42.431	[M − H]^−^	262.0173	261.01	261.0103	181, 97	1.1	QLD6
88	2,3‐Dihydroxy‐1‐guaiacylpropanone	C_10_H_12_O_5_	68.427	[M − H]^−^	212.0692	211.0619	211.062	167, 123, 105, 93	0.5	^a^QLD2, QLD3, QLD4, WAU5, WAU7, WAU10, WAU15, WAU 32
Curcuminoids										
90	Demethoxycurcumin	C_20_H_18_O_5_	56.969	^b^[M − H]^−^	338.1121	337.1048	337.1047	217	−0.3	^a^WAU10, WAU15, WAU17, WAU32
91	Curcumin	C_21_H_20_O_6_	60.583	^b^[M − H]^−^	368.1226	367.1153	367.1153	217	0.0	^a^WAU5, WAU7, WAU10, WAU15, WAU17, WAU20, WAU32
92	Bisdemethoxycurcumin	C_19_H_16_O_4_	65.359	[M + H]^+^	308.1041	309.1114	309.111	291, 263	−1.3	WAU15
Tyrosols										
93	Demethyloleuropein	C_24_H_30_O_13_	11.644	^b^[M + H]^+^	526.1669	527.1742	527.1742	495	0.0	^a^QLD2, QLD4, QLD5, QLD6, QLD7, WAU5, WAU7, WAU10, WAU15, WAU17, WAU20
Phenolic terpenes										
94	Epirosmanol	C_20_H_26_O_5_	17.762	[M + H]^+^	346.1772	347.1845	347.1847		0.6	^a^QLD4, WAU5
Hydroxyphenylpropenes										
95	Eugenol	C_10_H_12_O_2_	68.08	^b^[M − H]^−^	164.0831	163.0758	163.0755		−1.8	^a^WAU5, WAU20
Other polyphenols										
96	Salvianolic acid B	C_36_H_30_O_16_	57.92	[M − H]^−^	718.154	717.1467	717.1452	519, 339, 321, 295	−2.1	WAU5
97	Salvianolic acid C	C_26_H_20_O_10_	65.311	[M − H]^−^	492.1074	491.1001	491.1012	311, 267, 249	2.2	QLD2
Hydroxycoumarins										
98	Scopoletin	C_10_H_8_O_4_	13.489	[M − H]^−^	192.0414	191.0341	191.0341	174	0.0	^a^QLD1, QLD2, QLD3, QLD5, QLD6, WAU5, WAU10, WAU15, WAU17, WAU20
Hydroxybenzaldehydes										
99	Vanillin	C_8_H_8_O_3_	69.298	^b^[M + H]^+^	152.0459	153.0532	153.0532	136,122	0.0	^a^QLD5, QLD6, WAU5, WAU7, WAU10, WAU17
Stilbenes										
100	Resveratrol 5‐*O*‐glucoside	C_20_H_22_O_8_	32.334	[M − H]^−^	390.1305	389.1232	389.1225	227	−1.8	^a^WAU7, WAU15
101	4‐Hydroxy‐3,5,4′‐trimethoxystilbene	C_17_H_18_O_4_	58.99	^b^[M + H]^+^	286.1211	287.1284	287.1293	271, 241, 225	3.1	^a^QLD3, QLD5, QLD6, WAU15
102	3′‐Hydroxy‐3,4,5,4′‐tetramethoxystilbene	C_17_H_18_O_5_	68.956	^b^[M + H]^+^	302.1146	303.1219	303.1218	229, 201, 187, 175	−0.3	^a^QLD3, QLD6, WAU17
Lignans										
103	7‐Hydroxymatairesinol	C_20_H_22_O_7_	9.447	^b^[M + H]^+^	374.1376	375.1449	375.1451	343, 313, 298, 285	0.5	^a^QLD1, QLD2, QLD 3, QLD4, WAU15
104	Schisandrin C	C_22_H_24_O_6_	50.981	^b^[M − H]^−^	384.1595	383.1522	383.1527	370, 315, 300	1.3	^a^QLD1, QLD5, QLD6, QLD7, WAU5, WAU20
105	Schisandrin	C_24_H_32_O_7_	55.146	^b^[M − H]^−^	432.2136	431.2063	431.2064		0.2	^a^WAU10, WAU15, WAU20, WAU32
106	Episesamin	C_20_H_18_O_6_	56.537	^b^[M − H]^−^	354.108	353.1007	353.099		−4.8	^a^QLD2, QLD5, QLD6, WAU7, WAU10, WAU15, WAU17, WAU20, WAU32
107	7‐Oxomatairesinol	C_20_H_20_O_7_	59.041	^b^[M + H]^+^	372.1204	373.1277	373.1276	358, 343, 328, 325	−0.3	^a^QLD4, QLD6, QLD7, WAU5, WAU 10, WAU15, WAU20, WAU32
108	Schisandrol B	C_23_H_28_O_7_	60.35	^b^[M − H]^−^	416.1822	415.1749	415.174	224, 193, 165	−2.2	QLD2, WAU5
109	Enterolactone	C_18_H_18_O_4_	65.059	[M + H]^+^	298.1217	299.129	299.1291	281, 187, 165	0.3	WAU5
110	Pinoresinol	C_20_H_22_O_6_	67.031	^b^[M + H]^+^	358.1407	359.148	359.1473	342, 327, 313, 221	−1.9	^a^QLD1, QLD2, QLD3, QLD5, QLD 7, WAU15, WAU10
111	Secoisolariciresinol‐sesquilignan	C_30_H_38_O_10_	67.814	[M − H]^−^	558.2486	557.2413	557.2419	539, 521, 509, 361	1.1	QLD4

Abbreviations: QLD, samples from Queensland; RT, retention time; WAU, samples from Western Australia.

^a^ Compound was detected in more than one sample. Data presented in this table are from asterisk sample.

^b^ Compounds were detected in both negative [M − H]^−^ and positive [M + H]^+^ mode of ionization, while only single‐mode data are presented.

#### 3.4.1 Phenolic Acids

Thirty‐two phenolic acids were detected including hydroxybenzoic acids (10), hydroxycinnamic acids (19), hydroxyphenylacetic acid (1), and hydroxyphenylpropanoic acids (2).

Compound **3** was detected as gallic acid, as evidenced by the precursor ion [M − H]^−^ observed at *m/z* 169.0157. Further confirmation was achieved through MS/MS analysis, which revealed a peak fragment at *m/z* 125 resulting from the loss of a CO_2_ unit (44 Da) (Chou et al. 2021). Gallic acid is one of the most significant phenolic acids present in moringa pods and leaves (Amaglo et al. 2010). Gallic acid can neutralize free radicals that can cause cellular damage, potentially reducing the risk of chronic diseases (Kerdsomboon, Chumsawat, and Auesukaree 2021). Gallic acid may exhibit a wide range of biological activities, including anti‐inflammatory, anti‐tumor, antiviral, and antimicrobial activities (Prakash et al. 2007).

Compound **4** was identified to be paeoniflorin based on the detected *m/z* of 497.1562 in negative mode, which was subsequently verified through an MS/MS analysis, revealing the consecutive elimination of CH_2_O (30 Da) and benzoic acid (122 Da) (Liu, Agar, and Imran 2024). Paeoniflorin is a bioactive constituent commonly found in plants, which has been the subject of extensive research because of its favorable pharmacological properties. It has been demonstrated that paeoniflorin possesses antioxidant properties and exerts various bioactive functions. Its pharmacological effects include anti‐inflammatory, anti‐thrombotic, and immunomodulatory activities, rendering it a compound of significant interest for therapeutic applications (Zhou et al. 2020).

Compound **6** was identified as benzoic acid in both positive and negative ionization modes, with a tentative identification based on the precursor ion [M − H]^−^ observed at *m/z* 121.0302. The peak fragmentation at *m/z* 77 [M − H]^−^ indicates a loss of CO_2_ (44 Da), further supporting its identification as benzoic acid. This compound was detected in most samples from both regions (QLD1, QLD2, QLD4, QLD5, QLD6, and QLD7 and WAU5, WAU7, WAU10, WAU15 and WAU32).

Compound **7**, identified as ellagic acid, was detected in negative ionization mode with a precursor ion observed at *m/z* 301.0007. This identification was corroborated by the fragments observed at *m/z* 257 in MS/MS analysis, indicating a loss of CO_2_ (44 Da) from the precursor ion. El‐Shehawi et al. (2021) also reported the presence of this substance in moringa leaves. Shakeri, Zirak, and Sahebkar (2018) reported that ellagic acid possesses antioxidant energy, anticancer potential, and hepatoprotection activity. Rauha et al. (2000) also reported the antimicrobial activity of ellagic acid.

Compound **18**, detected in negative ionization mode, was identified as caffeic acid with a precursor ion at *m/z* 179.0368. Further confirmation of the compound was achieved through MS/MS analysis, which revealed product ions at *m/z* 143 and *m/z* 135, indicating the loss of 2 units of H_2_O and CO_2_, respectively. A study by Asgari‐Kafrani, Fazilati, and Nazem (2020) demonstrated that caffeic acid may play a role in reducing triglycerides and LDL cholesterol levels.

Ferulic acid, identified as compound **19**, was detected using mass spectrometry in positive ionization mode with a precursor ion at *m/z* 193.0519. Confirmation through MS/MS analysis revealed fragment peaks at *m/z* 178 and 194. Ferulic acid is noted as one of the most prevalent phenolic acids (Stohs and Hartman 2015), possessing the capability to enhance antioxidant enzymes, inhibit the formation of ROS, and scavenge free radicals.

#### 3.4.2 Flavonoids

Flavonoids are secondary metabolites, characterized by their structure as plant polyphenol molecules that consist of two benzene rings. Flavonoids represent the primary polyphenol compounds in moringa (Oldoni et al. 2019). Samples from both regions demonstrated a broad spectrum of flavonoids, including dihydrochalcones (3), flavanols (7), flavanones (7), flavones (22), and isoflavonoids (12).

8‐Prenylnaringenin (compound **51**) was detected under positive ionization mode. The compound exhibited a precursor ion at *m/z* 341.1383 and distinctive product ions at *m/z* 285. 8‐Prenylnaringenin is a flavonoid compound found in hops and is an essential ingredient in brewing beer, and is gaining interest because of its potential bioactivity, especially estrogenic effects (Paoletti et al. 2009). This constituent was widely detected in samples originating from QLD.

Compound **63** was recognized as Scutellarin with a precursor ion at [M − H]^+^ *m/z* 287.0554, featuring a distinctive fragment marked by the loss of an O_2_ unit (32 Da). Scutellarin, known for its strong antioxidant properties, was detected in samples from both QLD and WAU. Scutellarin has been widely studied as a natural medicine and has been experimentally shown to be helpful in the treatment of heart disease (Gao and Gu 2006).

Compound **65** was tentatively identified in a negative ionization mode with a precursor ion at *m/z* 446.0869. An MS/MS analysis revealed product ions at *m/z* 103 and *m/z* 163, indicating the presence of Kaempferol 7‐O‐glucoside owing to the loss of a glucose unit (284 Da). This compound has demonstrated antiviral properties as reported by Gansukh et al. (2016).

Quercetin 3′‐O‐glucuronide (compound **66**) was detected in both positive and negative ionization modes, with a precursor ion observed at *m/z* 479.0838. The confirmation of its identity was established through MS/MS analysis, where a peak fragment at *m/z* 301 was observed, indicating the loss of a glucuronic acid moiety (C_6_H_10_O_7_), which is commonly attached to flavonoids through glucuronidation (Zhu et al. 2022). This substance, reported in both wine and lotus flowers, is believed to exhibit sedative, anticonvulsant, and anxiety‐relieving properties (Kim et al. 2021).

Compound **68** was identified as Myricetin 3‐O‐galactoside with precursor ion at both negative and positive ionization modes with *m/z* at 479.0802. Myricetin 3‐*O*‐galactoside is a flavonoid glycoside derived from the flavonol myricetin and was only identified in samples from WAU, and it was reported as an active compound with medicinal potential (Xu et al. 2020). This compound has exhibited antioxidant, anti‐inflammatory, and antigenotoxic properties (de Oliveira Azevedo et al. 2015).

#### 3.4.3 Other Polyphenols

A total of 13 other polyphenol compounds were detected in moringa samples grown in Australia. These compounds were 1 Alkylphenol, 2 Hydroxybenzoketones, 3 Curcuminoids, 1 Tyrosol, 1 Phenolic terpene, 1 Hydroxyphenylpropene, 2 other polyphenols, 1 Hydroxycoumarin, and 1 Hydroxybenzaldehyde.

In the negative ion mode ([M − H]^−^), compound **98** was identified tentatively as a component of the hydroxycoumarins group, specifically scopoletin, across samples from both studied regions. The characterization of scopoletin was achieved with mass‐to‐charge ratios recorded as *m/z* = 191.0341 and MS/MS analysis confirmed it by the peak fragment at *m/z*174 because of loss of H₂O (18 Da). Scopoletin, also recognized as 7‐hydroxycoumarin, is a natural coumarin derivative commonly found in various plants. It is an aromatic compound known for its diverse biological activities. Scopoletin exhibits antioxidant, antimicrobial, anti‐inflammatory, and anticancer properties, highlighting its potential therapeutic applications (Jamuna et al. 2015).

Vanillin (compound **99**) was identified in both negative and positive ionization modes and was tentatively identified with at *m/z* 153.0532. This substance was detected in samples from both WAU and QLD. In support of this observation, this compound has been reported to be present in (Bhattacharya et al. 2018) and is thought to contribute to the antioxidant capacity of moringa.

#### 3.4.4 Stilbenes and Lignans

Three stilbenes and nine lignans derivatives were identified in the moringa pod samples. Compound **100** only observed in WAU samples with a precursor ion at 389.1225 was tentatively identified as Resveratrol 5‐*O*‐glucoside. Compounds 104 and 105 were detected as schisandrin derivatives in the negative model at *m/z* 383.1527 and 431.2064, respectively, presented in both two regions. Compound **106** with [M − H]^−^ at *m/z* 353.099 was tentatively identified as Episesamin, which was present in both QLD and WAU samples.

### 3.5 Venn Graphing of Polyphenol Compounds Distribution

**FIGURE 2:**
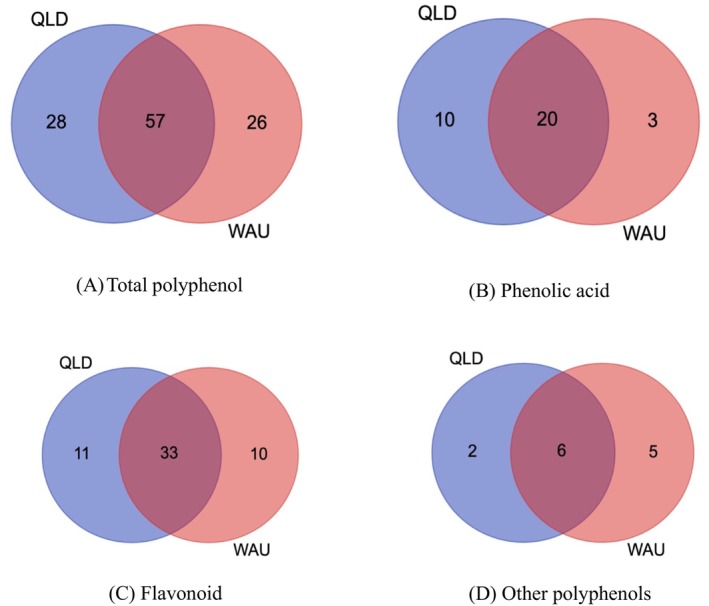
Venn diagram depicting the distribution of polyphenol compounds among moringa pod samples collected from two regions in Australia. Panel (A) illustrates the overlap of total polyphenol compounds across moringa pod samples from different regions. Panel (B) shows the correlation of phenolic acids among these samples. Panel (C) displays the relationship between flavonoids. Panel (D) highlights the connections of other polyphenol compounds within the moringa samples.

The Venn diagram provided a visualized representation of the distribution and overlap of antioxidant components in the moringa pod samples from QLD and WAU. An analysis of LC‐ESI‐QTOF‐MS/MS data showed distinct variations in the polyphenol profiles between QLD and WAU (Figure 2). The QLD samples exhibited a higher diversity of total polyphenol compounds than WAU samples, with 28 unique compounds identified in QLD and 26 in WAU, while 111 compounds were shared between both regions. These regional differences in polyphenol compound profiles can be attributable to the difference in environmental factors, such as soil composition, climate, and agricultural practices in QLD and WAU, potentially influencing the biosynthesis of polyphenols in moringa pods.

Figure 2B presents the profile of phenolic acids in moringa samples from both regions. QLD samples showed a greater variety of phenolic acids, with 30 compounds identified, compared to 23 phenolic acids identified in WAU samples. Notably, 20 phenolic acids were shared between samples from both regions. As illustrated in Figure 2C, 54 flavonoids were detected in QLD samples versus 43 flavonoids identified in WAU samples. Among them, 33 flavonoids were shared between samples from both regions. Samples originating from QLD were found to contain 8 other phenolic compounds, while 11 were identified in WAU samples, with 6 of these compounds being similar between both regions (Figure 2D).

## 4 Conclusions

This study identified significant differences in polyphenol compounds and antioxidant properties of moringa pods sourced from Queensland and Western Australia, with samples originating from Queensland showing higher TPC, TFC, and TCT. Seven different methods were used to detect antioxidant properties. LC‐ESI‐QTOF‐MS^2^ analysis identified 111 compounds, which included phenolic acids, flavonoids, and other polyphenols, with polyphenol types being more abundant in samples from Queensland. Overall, these findings demonstrate that moringa pods could be viewed as a rich source of natural antioxidants with high antioxidant capacity for developing functional foods. More experiments are warranted to assess Australia's most suitable growing environment targeting accumulation of polyphenol compounds in moringa pods.

## Author Contributions

**Rongjia Xie:** formal analysis (equal), investigation (equal), methodology (equal), visualization (equal), writing – original draft (equal). **Eric N. Ponnampalam:** investigation (equal), project administration (equal), resources (equal), writing – review and editing (equal). **Farhad Ahmadi:** investigation (equal), supervision (equal), writing – review and editing (equal). **Frank R. Dunshea:** resources (equal), validation (equal), writing – review and editing (equal). **Hafiz A. R. Suleria:** conceptualization (equal), funding acquisition (equal), methodology (equal), resources (equal), supervision (equal), validation (equal), writing – review and editing (equal).

## Ethics Statement

This study does not involve any animal or human experimentation. Moringa pods were collected from two farms in the Queensland and Western Australian regions.

## Data Availability

Data is available for sharing upon request.
